# Blood Transfusion Vs. Hydroxyurea for Stroke Prevention in Children With Sickle Cell Anemia: A Systematic Review and Meta-Analysis

**DOI:** 10.7759/cureus.31778

**Published:** 2022-11-22

**Authors:** Tamara A Hafiz, Sarah S Aldharman, Ruby N AlSubaie, Lena D Alzahrani, Ibrahim Ahmed J Albalawi, Yara A Alali, Maisaa M Yousuf, Hayat M Alharbi, Nawaf S Alamri, Syed F Jamil

**Affiliations:** 1 Public Health & Informatics, Umm Al-Qura University, Makkah, SAU; 2 College of Medicine, King Saud Bin Abdulaziz University for Health Sciences, Riyadh, SAU; 3 College of Medicine, University of Tabuk, Tabuk, SAU; 4 Collage of Medicine, King Saud Bin Abdulaziz University for Health Sciences, Jeddah, SAU; 5 Research, King Abdullah International Medical Research Center, Riyadh, SAU; 6 College of Medicine, King Abdulaziz University for Health Sciences, Riyadh, SAU; 7 Pedaitrics, King Abdullah Specialized Children's Hospital, Riyadh, SAU

**Keywords:** children, prevention, stroke, sickle cell anemia, hydroxyurea, blood transfusion

## Abstract

Sickle cell anemia (SCA) is a hereditary condition that can lead to severe complications in children such as acute coronary syndrome, splenic sequestration, renal failure, and stroke. Blood transfusion and hydroxyurea (HU) therapy are used to prevent stroke in children with sickle cell disease (SCD). Preliminary data show considerable variation and inconsistency in the use of these two therapeutic interventions. Therefore, this systematic review was carried out to compare the effects of blood transfusion to HU therapy in preventing stroke for children with sickle cell disease. There was an extensive literature search in reliable and authentic databases like PubMed, Medline, Scopus, Cochrane, and Science Direct to obtain relevant articles. This study used the standards and guidelines from the Preferred Reporting Items for Systematic Reviews and Meta-Analyses (PRISMA). During the systematic review, data were obtained focusing on the following parameters: the size of the sample in the study, the age of the subjects involved in the study, the type of Intervention, and the outcome. After an initial search of 163 papers, 25 studies were included. The results of the research give the first evidence that HU is effective in the treatment of cerebrovascular problems in children with SCD. However, it is unclear under what circumstances HU may prevent a second stroke. It can be concluded that children with SCA can effectively avoid primary strokes through chronic blood transfusions and HU.

## Introduction and background

Sickle cell anemia (SCA) is a hereditary condition that increases the risk of cerebral vasculopathy, which can result in strokes, especially in children which exposed them to a high-risk category for developing a stroke. Stroke is among the most severe clinical consequences that can affect children with sickle cell disease (SCD) and is a major cause of mortality in this patient group [1-3]. A stroke occurs in around 5%-10% of children with SCD, most frequently in their first decade of life. The recurrence of stroke was observed in 46% of children with SCD at an average interval of nine months according to the transcranial Doppler (TCD), which detects patients at risk of strokes [4,5]. Patients who were observed as having high cerebral velocities (>200 cm/s) were considered abnormal and had a risk of 40% for stroke to occur within three years after detection [4-7]. However, people with normal cerebral velocities (<170 cm/s) had a 2% or less risk of stroke. Blood transfusions and hydroxyurea (HU) play the main role in the prevention of stroke in children with SCA. In individuals with SCD, transfusion treatment has been demonstrated to be useful in preventing both main and secondary strokes [8]. Specific requirements determine the choice for either using HU therapy or blood transfusion. According to international standards, individuals with an acute ischemic stroke should be treated with exchange transfusions with the aim of achieving hemoglobin (Hb) of 10 g/dL and HbS of <30% [8]. Also, treatment for SCD patients is mostly supportive with HU serving as the only widely prescribed medication that alters the etiology of the illness. By raising fetal hemoglobin (HbF), it enhances clinical outcomes and lowers the likelihood of "sickling" episodes [9]. HU therapy helps reduce the incidences of blood transfusion, acute chest syndrome, acute pain, and the mortality of SCD patients [10]. It is a useful alternative for regular blood transfusion for SCD [11].

Many pediatric patients with SCD and stroke have persistent physical and neuropsychological impairments [12-14]. Nine out of 10 (90%) untransfused juvenile patients with SCD and stroke experienced a recurrent stroke [15]. Transfusions can be given as a simple transfusion or as an exchange transfusion. The goals of transfusion in SCD are to enhance oxygen-carrying capacity while also decreasing the fraction of sickle hemoglobin (HbS) relative to hemoglobin A (HbA) to avoid or reverse vaso-occlusion consequences. In acute conditions, simple transfusion will enhance oxygen-carrying capacity. Exchange transfusion provides the benefit of both raising oxygen-carrying capacity and lowering HbS% [16,17]. In individuals on long-term transfusion, both repeated simple or exchange transfusions can maintain a low HbS%. Simple transfusion is the most frequently used type of transfusion in chronic transfusion programs in children with the need for iron chelation therapy after approximately one year of transfusion [16,17]. After terminating a short-term (1-2 years) transfusion regimen, a 70% recurrence rate was reported, and a 50% recurrence rate was observed after terminating a long-term (5-12 years) transfusion regimen [18,19]. Therefore, most juvenile hematologists recommend continuous chronic transfusions to minimize recurrent strokes, even with the long-term potential consequences of transfusions, which include the possibility of the transfer of infectious organisms, erythroid alloimmunization, and iron overload. Even though transfusion treatment is effective, some patients are unable to continue it. For instance, there is a report of two young individuals with SCD and stroke who were incapable of continuing their chronic transfusion treatment and had to stop receiving transfusions. Then, as a preventative measure against stroke recurrence, the patients received oral HU therapy [20]. A Phlebotomy regimen was adopted to minimize iron excess and promote endogenous erythropoiesis. Both patients reacted favorably to the HU treatment and neither patient experienced stroke recurrence during nearly three years of therapy. Based on this testimonial success, blood transfusions were terminated prospectively in a new and bigger sample of children with SCD and stroke in another report [4]. The findings show that some children with SCD and stroke might be able to stop receiving chronic transfusions and instead utilize daily oral HU treatment as stroke prevention. Although much of the morbidity related to chronic blood transfusion treatment has been decreased because of better testing of blood units for pathogenic pathogens, iron overload is still an important long-term issue [21]. Long-term cohort research showed that switching from continuous transfusion to HU with follow-up can be safe in some patients [22]. An advantage of HU is its potential to reduce the high white blood cell counts linked with early mortality, stroke, and acute chest syndrome in SCA [23].

Therefore, searching for effective alternatives and evidence regarding the clinical efficacy of HU in comparison to blood transfusion in pediatric patients with SCD is warranted to overcome such an issue. Preliminary findings demonstrate a significant variation and inconsistency in the use of these two therapy techniques. Limited studies have been conducted to address preventive options for stroke associated with SCD. Therefore, this study sought to compare the effects of blood transfusion to HU therapy in preventing stroke for children with SCD.

## Review

Methodology

Study Design

This study used the standards and guidelines from the Preferred Reporting Items for Systematic Reviews and Meta-Analyses (PRISMA). The study was prepared using PRISMA extensions published in the Cochrane Handbook for Systematic Reviews of Interventions [24]. 

Search Strategy

Cochrane, PubMed, Medline, Scopus, and Science Direct were used as electronic databases to identify relevant articles for the study. The search strategy utilized keywords and their combinations, field tags, truncations, and Boolean operators "AND" and "OR." Search strings were used to ensure that an accurate scope of the materials required was covered effectively. The Mesh used in the study included "Anemia, Sickle Cell/prevention and control"[Mesh] OR "Anemia, Sickle Cell/therapy" [Mesh], "Stroke/blood" [Mesh] OR "Stroke/prevention and control" [Mesh] OR "Stroke/therapy" [Mesh], "Blood Transfusion/blood" [Mesh] OR "Blood Transfusion/therapy" [Mesh], "Hydroxyurea/administration and dosage" [Mesh] OR "Hydroxyurea/therapeutic use" [Mesh]. The studies identified were put together for search on their eligibility.

Eligibility Criteria

The researchers agreed upon the eligibility criteria and guidelines for the study to ensure the best articles were identified. Articles selected had to pass through the PICOS criteria (Population, Intervention, Comparison, and Outcomes). The population needed for the study had to be children. The intervention for the study was blood transfusion, the control was HU therapy, and the outcome was stroke prevention. Included articles had to have the participating children between one and 15 years old. Studies that were not related to children were excluded. Included articles had to have been published in English or translated to English. Unpublished articles were not selected as part of the research study. Articles that had written about other medicines and pharmaceutical compounds other than hydroxycarbamide or HU were excluded.

Quality Assessment

A quality appraisal for the selected studies was performed using AXIAL criteria. The quality assessment sections were divided into four. The first section was on the introduction and its validity on the aims and objectives of the study. Section 2 was on the validity of the methods for the included studies. Sections 3 and 4 covered the results and discussions, respectively. Answers for the assessment questions were categorized into yes, no, and not applicable, which were abbreviated to "Y," "N," and "NA," respectively. 

Data Extraction

The researchers used predesigned Excel worksheets in the extraction of data. The researchers extracted the authors, the year of publication, interventions, outcomes, and observed results. An analysis of the studies identified and the results got was done to ensure that there was unity. Whenever cases of dispute arose in any matter, a third party was used in the resolution process. 

Result Analysis

Two forms of analysis were adopted for this investigation. We used qualitative assessment (systematic review) and quantitative assessment (meta-analysis). Literal analysis was adopted to conduct a systematic review of the evidence provided by the included studies. Statistical analysis was conducted on STATA Version 17.0 (Stata V.17.0). The analysis determined the heterogeneity between the included studies using the T^2^, I^2^, and H^2^ statistics. A level of heterogeneity between 25%<I^2^< 50% and below was considered more fitting. In the meta-analysis, the investigation adopted a random-effects model to find the odds ratio to determine the effect size of the outcomes. The P-values shall determine the test's significance; significance shall be attained if the P-value ≤0.05. The results of this meta-analysis were represented in various forest plots. Funnel plots were also generated to show the symmetry of the included studies and represent the studies' publication bias to give credence to the relationships created.

Results

Study Selection

**Figure 1:**
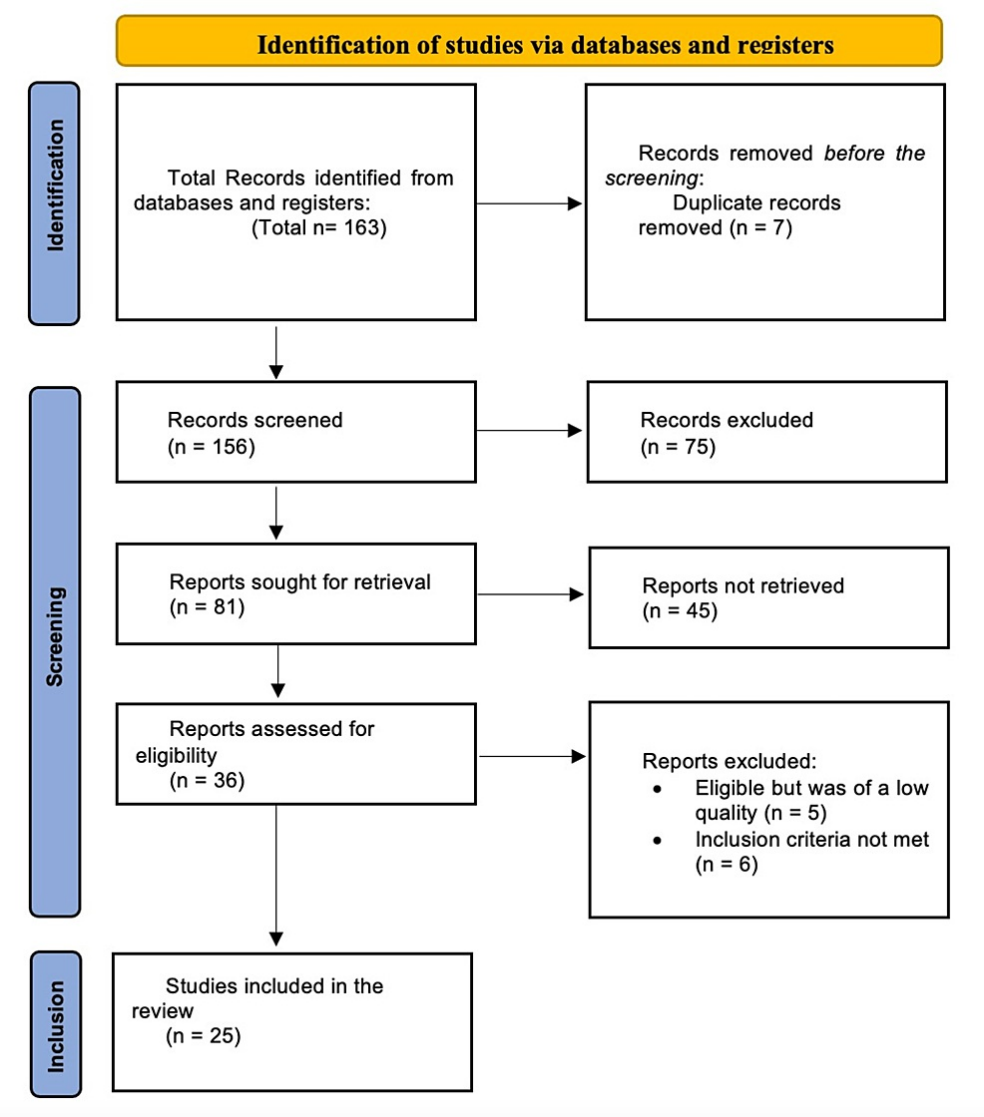
PRISMA flow diagram of the inclusion and exclusion process of the studies in the systematic review PRISMA, Preferred Reporting Items for Systematic Reviews and Meta-Analyses.

The study search conducted in the electronic databases identified 163 studies. These studies were identified using the search strings. Seven articles were excluded as they were duplicates remaining with 156 studies. A screening process was then conducted by sorting through the titles, abstracts, and the outcomes of interest. In this stage, 75 records were excluded remaining 81 articles. Finally, records were sought for retrieval, and only 36 were retrieved, excluding 45 records. Five studies were excluded because of having low-quality data and six were excluded for failing to meet the inclusion criteria. Twenty-five studies were identified and used for the systematic review and meta-analysis. Figure 1 represents a PRISMA flowchart diagram that shows the study selection process.

The characteristics of the included studies are shown in Table 1.

**Table 1:** The characteristics of the studies ACS: acute coronary syndrome; MCV: mean corpuscular volume; HU: hydroxyurea; HbS: sickle hemoglobin; HbF: fetal hemoglobin; TCD: transcranial Doppler; SCA: sickle cell anemia; TAMMV: time-averaged maximum mean velocities; MRI: magnetic resonance imaging; MRA: magnetic resonance angiography; MCA: middle cerebral artery; SCD: sickle cell disease.

Author	Demography	Intervention	Outcome
Ware et al. (1999) [4]	n = 16, Mean age = 7.1 ± 4.	Average HU dose of 24.9 ± 4.2 mg/kg/day; Phlebotomy for those with iron overload.	Hemoglobin concentration: 9.4 ± 1.3 g/dL; Mean corpuscular volume: 112 ± 9 FL; %HbF: 20.6% ± 8.0%; %F cells: 79.3% ± 14.7%.
Fridyland et al. (2017) [25]	2-year-old African American boy with sickle cell disease.	Partial exchange transfusion with a baseline HbS of 48.4%, HbC of 47.9%, HbF of 3.7%. HU 300 mg (22.2 mg/kg/day).	Total hemoglobin increased from 9.4 to 11.3 g/dL. MCV from 78.6 to 90.9 FL. HbF from 3.7% to 5.1%. No recurrence of clinical symptoms for two years.
Darshana et al. (2021) [26]	A review of 41 papers	HU and blood transfusion therapies for SCD in South Asia.	HU therapy: 14 prospective trials. Most (n = 10; 71.4%) adopted fixed low dose of 10 mg/kg/day regimen. HU therapy: significant reductions in vaso-occlusive crises and transfusion requirements.
Abboud (2020) [27]	A review of the standard management of SCD complications.	Transfusions, HU, and new therapeutic agents in managing SCD complications such as pain, stroke, and acute chest syndrome.	Survival rate: increased in children and adolescents in developed countries. Adults’ survival rate: lagged. Pediatric survival rate increase: due to HU treatment and stroke prevention strategies.
Bernaudin (2016) [22]	92 patients. Children from the CHIC newborn cohort with SCA (abnormal time averaged maximum mean velocities [TAMMV] ≥200 cm/s). Mean age of 3.7 years (range, 1.3-8.3 years).	Normalized TCD on transfusions safely switched to HU treatment.	83.5% of patients: normalization of velocities (TAMMV < 170 cm/s). Stenosis: 27.5% of patients was associated with the risk of non-normalization (P < 0.001). Reversion: 13/45 patients (28.9%). Transplant: 24 patients; it allowed transfusions to be safely stopped in all patients and velocities normalized in 4 patients.
Transfusion			
Aygun et al. (2011) [28]	340 children with abnormal TCD velocities.	Chronic transfusion.	Mean pre-transfusion HbS: 33.2 ± 14.0%, 75th percentile: 41% HbS, 90th percentile: 50% HbS.
Aygun et al. (2009) [29]	295 children with SCA and stroke. Mean age: 11.9 vs 12.0 years.	Transfusions changing to HU.	Average pre-transfusion %HbS: 35 ± 11%, 75th percentile: 43% HbS, 90th percentile: 52% HbS.
Brousse et al. (2009) [30]	18 patients. Blood flow velocity ≥200 cm/s.	Chronic transfusions.	Baseline (MRI + MRA): 10 (2-22); follow-up (MRI + MRA): 12 (3-26) (P = 0.027).
Mirre et al. (2010) [31]	29 children. Mean age: 6.8 ± 3.0 years.	Chronic transfusion.	Stroke recurrence rate: 1.6/100 patient-years. Mean HbS levels before: 30 ± 10%. Mean HbS levels after: 20.6 ± 7%.
Moreira Franco et al. (2020) [32]	n = 15. Mean age: 118.67 ± 41.40 months.	Chronic transfusion	Before mean HbS: 75.18 ± 11.69%. After 24 months mean HbS: 43.78 ± 10.6%. TCD velocity in 12 months: 158.5 ± 28.89 cm/s (right) and 157.62 ± 34.43 cm/s (P = 0.02). In 24 months 149.63 ± 26.95 cm/s (right) and 143.7 ± 32.27cm/s.
Wood et al. (2016) [33]	121 participants. Mean age: 9·8 ± 2·9 years.	Chronic transfusions.	Liver iron concentration: 9.0 ± 6.6 mg/g dry weight. Serum ferritin: elevated to 2696 ± 1678 lg/L. Spleen R2*: 509 ± 399 Hz. Pancreas R2*: increased in 38.3%. Kidney R2*: increased in 80.7%.
Hydroxyurea			
DeBaun (2020) [11]	110/120 of children with SCA. Mean age: >5 years.	Initial low dose of HU: 10 mg/kg per day	TCD: Lowers to <200 cm/s in the majority of children. Low-dose HU: has a negligible risk for myelosuppression.
DeBaun et al. (2014) [34]	269 participants. Age: 5-12 years with hemoglobin SS or SB0 thalassemia, at risk of developing stroke, with a high TCD velocity in the MCA ≥200 cm/s	Low-dose HU therapy: 20 mg/kg/day for 36 months.	One child on HU therapy was hospitalized for 5 days for hypovolemia and dehydration associated with cholera. Increase in MCV from baseline to 3 months after starting HU therapy with a minimum increase in MCV of at least 3 Fl in 8 of 11 participants.
Ware (2010) [35]	84 school-aged children with severe clinical manifestations. Median age: 9.1 years.	Open-label HU	Average HbF: 20%. Hospital admissions for pain and other vaso-occlusive episodes decreased.
Mulaku et al. (2013) [36]	A systematic review of systematic reviews (n = 19 studies).	A summary of the available evidence on the efficacy, effectiveness, and safety of HU in the management of SCD in children below 5 years.	Risk of stroke recurrence: 2/100 vs 20/33 in control. TCD values decreased with average reduction of 25.6 ±27.6 cm/s. HU resulted in a signiﬁcant decrease in TCD velocity in the right MCA (166 ± 27 cm/s to 135 ± 27 cm/s).
Montalembert et al. (2006) [37]	225 SCD children. Mean age: 9.2 ± 4.4 years.	HU.	Mean hemoglobin levels: increased to 8.2 ± 1.5 g/dL from 6.6 ± 0.5 g/dL.
Núñez et al. (2020) [38]	A meta-analysis of 2 studies looking at a sum of 254 patients.	HU and chronic blood transfusion	Stroke occurrence: risk difference 0.04 (95% CI: 0.03-0.03). New-onset neurological deficit: risk difference 0.11 (95% CI: 0.00-0.21). Vaso-occlusive crisis: risk difference 0.10 (95% CI: 0.001-0.20).
Sumoza et al. (2002) [39]	5 children with SCA	HU dose: 40 mg/kg/day and one 30 mg/kg/day.	Hb contraction: 19.5 g/L. No stroke recurrence during the 42-112 months.
Bortolusso Ali et al. (2011) [40]	43 children	HU	Clinical risk of stroke recurrence: 2/100 person-years.
Mvalo et al. (2019) [41]	187 patients with SCD	HU at doses of 10-20 mg/kg/day	Decreases in the rates of hospitalization (−4.1 per 1000 person-days; −7.2, −1.0; P = 0.004). Transfusions (−2.3 per 1000 person-days; 95% confidence interval: −4.9,0.3; P = 0.06). Annual school absenteeism (−51.2 per person-years; −60.1, −42.3; P < 0.0001).
Cunningham-Myrie et al. (2015) [42]	43 children with SCD. 10 opted for HU therapy.	HU.	HU use led to decreased stroke recurrence and death without signiﬁcantly increasing the annual cost of care per patient (J$83,250 vs. J$76,901, P = 0.491).
Lefèvre et al. (2008) [43]	119 patients.	HU.	Concerning the secondary prevention of stroke, the recurrence rate of stroke in the patients treated with HU was 2.9 for 100 patient-years, similar to that recorded in chronically transfused patients. Concerning the primary prevention of stroke, the incidence of first stroke in our patients with HU was 0.36 for 100 patient-years.
Abdullahi et al. (2020) [44]	220 children (mean age: 7.5 years).	Low- and moderate- fixed-dose HU	Incidence rates of strokes per 100 person-years: 1.19 and 1.92 (low and moderate, respectively). Incidence rate ratio: 1.60. 95% CI: 0.31-10.34, P = 0.768.
Al Hawsawi and Turkistani (2008) [45]	10 patients. Age range: 5-15 years	15-30 mg/kg/day HU dose	Pain crisis: 70 episodes to 25; P-value <0.05. ACS: decreased from 8 to zero; P-value <0.025. 80% of patients showed a significant MCV increase after HU therapy. No adverse events occurred.
Ware et al. (2016) [46]	121 randomized participants (61 transfusions, 60 HU).	Transfusion and HU (mean 27 mg/kg/day).	TCD velocities: 143 ± 1.6 and 138 ± 1.6 cm/s (95% CI) = 4.54 (0.10, 8.98).
Hankins et al. (2014) [47]	28 infants enrolled in the original 2-year HUSOFT study.	Open-label liquid HU at 20 mg/kg/day for 15 years.	There were 5.1 vaso-occlusive events (pain and acute chest syndrome)/100 patient-years, 7.3 packed red blood cell transfusions/100 patient-years. No malignancies, strokes, or deaths occurred. At last follow-up, all subjects were at appropriate grade level (10-12 grade) with no history of repeated grades.
Galadanci et al. (2020) [48]	29 children with abnormal TCD	Fixed-dose HU (~20 mg/kg/day)	Intervention vs control stroke incidence rate: 0.76 per 100 person-years (95% CI: 0.11-5.24) vs 0.43 per 100 person-years (95% CI: 0.16-1.15).

Quality Analysis

The study's quality analysis was conducted using AXIAL. The analysis was grouped into introduction, method, results, and discussions. The answers to the quality assessment were yes, no, and not applicable, which were shortened to "Y," "N," and "NA." The AXIAL assessment appraisal results of the included studies are shown in Table 2.

**Table 2:** AXIAL assessment appraisal results of the included studies Included studies are Ware et al. [4], Bernaudin et al. [22], Fridyland et al. [25], DeBaun [11], DeBaun et al. [34], Ware [35], Mulaku et al. [36], Aygun et al. [28], Aygun et al. [29], Brousse et al. [30], Mirre et al. [31], Moreira Franco et al. [32], Wood et al. [33], Montalembert et al. [37].

Variable	Ware et al. (1999)	Bernaudin et al. (2016)	Fridyland et al. (2017)	DeBaun (2020)	DeBaun et al. (2014)	Ware et al. (2010)	Mulaku et al. (2013)	Aygun et al. (2011)	Aygun et al. (2009)	Brousse et al. (2009)	Mirre et al. (2010)	Moreira Franco et al. (2020)	Wood et al. (2016)	Montalembert et al. (2006)
Introduction														
Were clear aims and objectives of the study used?	Y	Y	Y	Y	Y	Y	Y	Y	Y	Y	y	y	Y	Y
Methods														
Was the study design appropriate for the stated aim of the study?	Y	Y	Y	Y	Y	Y	Y	Y	Y	Y	Y	Y	Y	Y
Was the sample size simplified?	Y	Y		Y	Y	Y	Y	Y	Y	Y	Y	Y	Y	Y
Was the target/reference population clearly defined? (Is it clear who the research was about?)	Y	Y	N	Y	Y	Y	Y	N	Y	Y	N	Y	Y	Y
Was the sample frame taken from an appropriate population base so that it closely represented the target/reference population under investigation?	Y	Y	Y	Y	Y	Y	Y	Y	Y	Y	Y	Y	Y	Y
Was the selection process likely to select subjects/participants that were representative of the target/reference population under investigation?	Y	N	Y	Y	Y	Y	Y	N	Y	Y	Y	Y	Y	N
Were measures undertaken to address and categorize non-responders?	Y	Y	Y	Y	Y	Y	Y	Y	Y	Y	N	Y	N	Y
Were the risk factor and outcome variables measured appropriate to the aims of the study?	Y	Y	Y	Y	Y	Y	Y	Y	Y	Y	Y	Y	Y	Y
Is it clear what was used to determine statistical significance and/or precision estimates? (e.g. P-values, confidence intervals)	Y	Y	N	N	N	Y	Y	Y	Y	N	Y	Y	Y	Y
Were the methods (including statistical methods) sufficiently described to enable them to be repeated?	Y	Y	Y	Y	Y	Y	Y	Y	Y	Y	Y	Y	Y	Y
Results														
Were the basic data adequately described?	Y	Y	Y	Y	Y	Y	Y	Y	Y	Y	Y	Y	Y	Y
Does the response rate raise concerns about non-response bias?	Y			Y	Y	Y	Y	Y	Y	Y	Y	Y	N	Y
If appropriate, was information about non-responders described?	Y			Y	Y	Y	Y	Y	Y	Y	N	Y	Y	Y
Were the results internally consistent?	Y	Y	Y	Y	Y	Y	Y	Y	Y	Y	Y	Y	N	Y
Were the results presented for all the analyses described in the methods?	Y	Y	Y	Y	Y	Y	Y	Y	Y	Y	Y	Y	Y	Y
Discussion														
Were the authors' discussions and conclusions justified by the results?	Y	Y	Y	Y	Y	Y	Y	Y	Y	Y	Y	Y	Y	Y

The AXIAL assessment appraisal results of the remaining included studies are presented in Table 3.

**Table 3:** AXIAL assessment appraisal results of the included studies (continued) Included studies are Núñez et al. [38], Sumoza et al. [39], Bortolusso Ali et al. [40], Mvalo et al. [41], Cunningham-Myrie et al. [42], Lefèvre et al. [43], Abdullahi et al. [44],  Al Hawsawi and Turkistani [45],  Ware et al. [46], Darshana et al. [26], and Abboud [27].

Variable	Núñez et al.(2020)	Sumoza et al. (2002)	Bortolusso Ali et al. (2011)	Mvalo et al. (2019)	Cunningham-Myrie et al. (2015)	Lefèvre et al.(2008)	Abdullahi et al. (2020)	Al Hawsawi and Turkistani (2008)	Ware et al. (2016)	Darshana et al. (2021)	Abboud (2020)
Introduction											
Were clear aims and objectives of the study used?	Y	Y	Y	Y	Y	Y	Y	Y	Y	Y	Y
Methods											
Was the study design appropriate for the stated aim of the study?	Y	Y	Y	Y	Y	Y	Y	Y	Y	Y	Y
Was the sample size simplified?	Y	Y	Y	Y	Y	Y	Y	Y	Y	Y	N
Was the target/reference population clearly defined? (Is it clear who the research was about?)	Y	Y	N	Y	Y	Y	Y	Y	Y	Y	Y
Was the sample frame taken from an appropriate population base so that it closely represented the target/reference population under investigation?	Y	Y		Y	Y	Y	Y	Y	Y	Y	Y
Was the selection process likely to select subjects/participants that were representative of the target/reference population under investigation?	Y	Y		Y	Y	Y	Y	Y	Y	Y	Y
Were measures undertaken to address and categorize non-responders?	Y	N		Y	Y	Y	Y	Y	Y	Y	Y
Were the risk factor and outcome variables measured appropriate to the aims of the study?	Y	Y		Y	Y	Y	Y	Y	Y	Y	N
Is it clear what was used to determine statistical significance and/or precision estimates? (e.g. P-values, confidence intervals)		N									Y
Were the methods (including statistical methods) sufficiently described to enable them to be repeated?	Y	Y	Y	Y	Y	Y	Y	Y	Y	Y	N
Results											
Were the basic data adequately described?	Y	Y	Y	Y	Y	Y	Y	Y	Y	Y	Y
Does the response rate raise concerns about non-response bias?	Y	Y	N	Y	Y	Y	Y	Y	Y	Y	N
If appropriate, was information about non-responders described?	Y	Y	Y	Y	Y	Y	Y	Y	N	Y	Y
Were the results internally consistent?	Y	Y	Y	Y	Y	Y	Y	Y	Y	Y	Y
Were the results presented for all the analyses described in the methods?	Y	Y	Y	Y	Y	Y	Y	Y	Y	Y	Y
Discussion											
Were the authors' discussions and conclusions justified by the results?	Y	Y	Y	Y	Y	Y	Y	Y	Y	Y	Y

Statistical analysis

Transfusion

**Figure 2:**
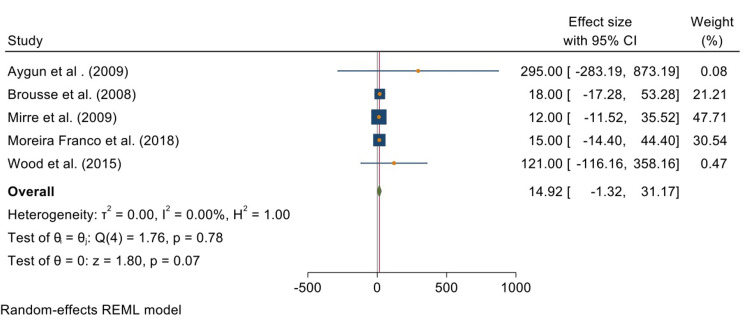
Forest plot summarizing the analysis of the effects of transfusion on stroke incidences in children with sickle cell anemia. Included studies are Aygun et al. [29], Brousse et al. [30], Mirre et al. [31], Moreira Franco et al. [32], and Wood et al. [33].

Five studies reported the effects of transfusion on reducing the incidence of stroke. The overall test results reveal a random effects size and odds ratio of 14.92 [-1.32, 31.17] at 95% CI. This meta-analysis shows no significant impact of transfusion on children with SCA (P = 0.07). The studies used to analyze this were not heterogeneous (T^2^ = 000; I^2^ = 0.00%; H^2^ = 1.00). Figure 2 is a forest plot summarizing this analysis.

**Figure 3:**
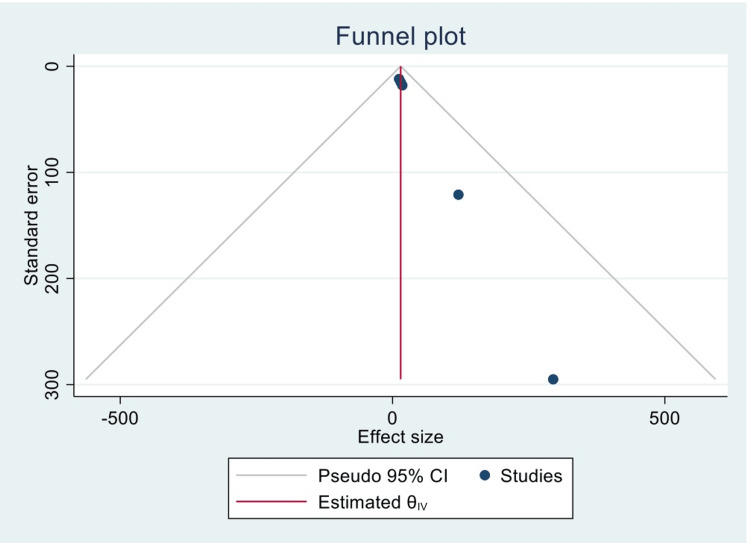
Funnel plot representing publication bias of the included and analyzed studies

Figure 3 is a funnel plot reporting the publication bias between the previous five studies.

Hydroxyurea

**Figure 4:**
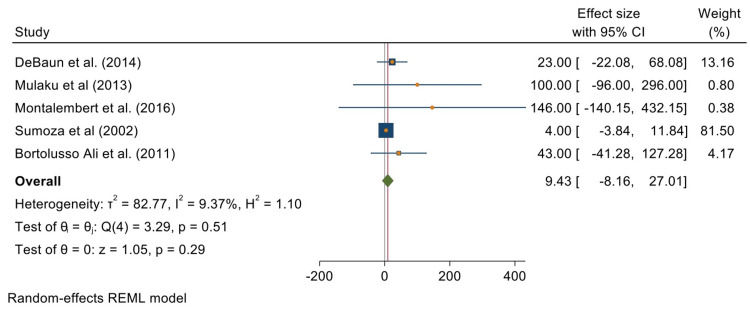
Forest plot summarizing the analysis of the effects of hydroxyurea on stroke incidences in children with sickle cell anemia Included studies are DeBaun et al. [34], Mulaku et al. [36], Montalembert et al. [37], Sumoza et al. [39], and Bortolusso Ali et al. [40].

Five of the 15 studies reporting on HU's effects on reducing stroke incidence provided data for a meta-analysis. The overall test results reveal a random effects size and odds ratio of 9.43 [-8.16, 27.01] at 95% CI. This meta-analysis shows no significant impact of HU on children with SCA (P = 0.29). The studies used to analyze this were not heterogeneous (T^2^ = 82.77; I^2^ = 9.37%; H^2^ = 1.10). Figure 4 is a forest plot summarizing this analysis.

**Figure 5:**
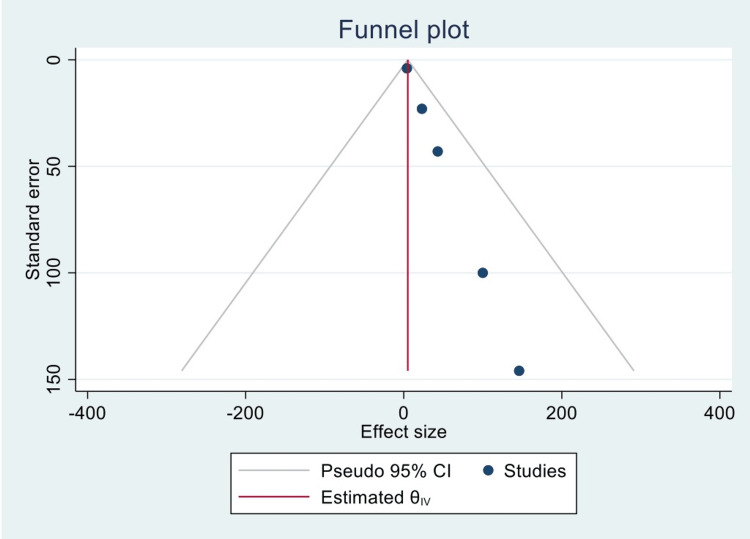
Funnel plot representing publication bias of the included and analyzed studies

Figure 5 is a funnel plot reporting the publication bias between the previous five studies.

Both (Transfusion and HU)

**Figure 6:**
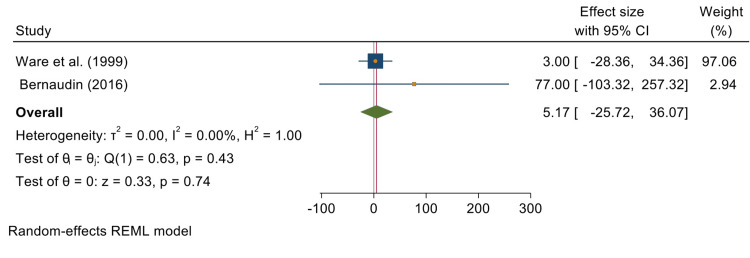
Forest plot summarizing the analysis of the effects of both transfusion and hydroxyurea on stroke incidences in children with sickle cell anemia Included studies are Ware et al. [4] and Bernaudin et al. [22].

Two of the five studies reporting on the effects of both transfusion and HU on reducing the incidence of stroke were used in this analysis. The overall test results reveal a random effects size and odds ratio of 5.17 [-25.72, 36.07] at 95% CI. This meta-analysis shows no significant impact of using both transfusion and HU on children with SCA (P = 0.74). The studies used to analyze this indicated a low level of heterogeneity (T^2^ = 0.00; I^2^ = 0.00%; H^2^ = 1.00). Figure 6 is a forest plot summarizing this analysis.

**Figure 7:**
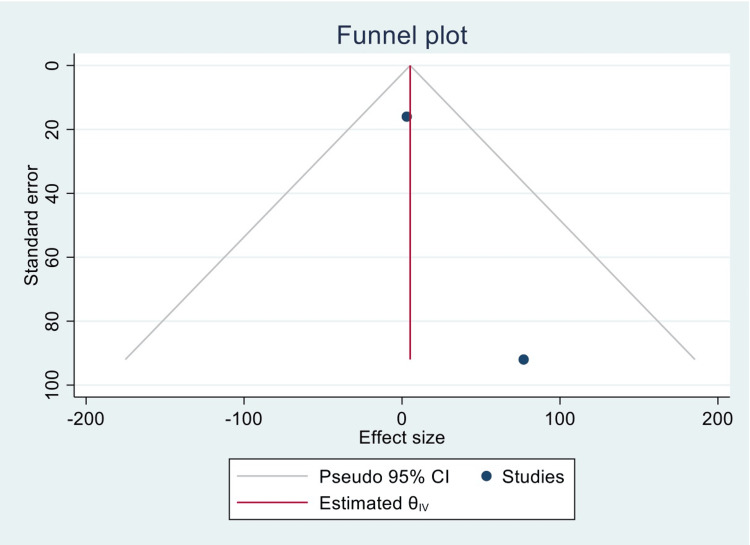
Funnel plot representing publication bias of the included and analyzed studies

Figure 7 is a funnel plot reporting the publication bias between the previous two studies.

Discussion

A stroke occurs in approximately 11% of children with SCA by the age of 20 years. The first episode occurs more frequently from 2-5 years old [39]. Approximately 50% of children with SCA and an initial stroke that do not receive chronic transfusions develop another stroke in the following three years. By comparison, only 10% of transfused patients have a reoccurring stroke [39]. Significant downsides of chronic transfusions are iron overload, which may lead to fatality or serious arrhythmias. It has also not been determined how much time they should be continued. Also, unidentified is the risk of reoccurring stroke after transfusions are terminated [39].

A preliminary information recommend HU therapy in some children with SCD as an alternative to blood transfusion for recurrent stroke prevention [4]. The increase in HbF criteria (%HbF and %F cells) achieved by HU is most likely important in avoiding in vivo sickling within the stenotic analytical vessels. Patients accomplished an average %HbF of approximately 20% and %F cells of approximately 80%, which could prevent intracellular sickling [4]. Additional feasible devices include reductions in total white blood cell matter and neutrophil matter with HU treatment. Moreover, improved rheological qualities of the erythrocytes brought about changes in erythrocyte morphology, adhesiveness, and cation material. Using the same HU dosage escalation schedule revealed that the Hb concentration, mean corpuscular volume (MCV), %HbF, and %F cells increase throughout the ﬁrst six months of HU treatment [4]. However, they continued to increase between six months and a year of treatment. The phlebotomy program was designed to lower the iron concentration and promote erythropoiesis, thereby boosting the variety of HbF-containing reticulocytes and preventing stroke reoccurrence. The phlebotomy regimen was very well tolerated, and most patients had 10 mL/kg of blood removed every two weeks while preserving a Hb concentration above 8 g/dL. Phlebotomy has led to constant reductions in the total body iron stores, with considerable diminution in the lotion ferritin [4]. HU could also be considered for the avoidance of primary stroke. TCD can recognize children with SCD with a boosted risk of primary stroke, and transfusion treatment can prevent primary stroke in scientific setups.

Considering the risk of serious adverse effects associated with chronic transfusion and the constant improvement with the time of the transplant outcomes with gene-identical donors, domestic human leukocyte antigen (HLA) inputting was performed, and the transplant was discussed with parents of children with SCD [22]. The study reported previously that participants transplanted since 2000 had a 95% chance of cure, a percent conﬁrmed in a French collection, with about a 3% risk of death. In the examined cohort, 24 of 92 participants were transplanted. Donor engraftment was effective, and chronic transfusion was done by all participants [22]. Moreover, quick normalization of velocities occurred in four participants that had irregular velocities before the transplant despite chronic transfusion. HU was efficiently re-introduced in six participants after the new normalization of chronic transfusion. The Kaplan-Meier (KM) estimate of the percentage of participants with irregular TCD imaging reversion on HU at one year was 19.2% (95% CI, 7-31.4), which is below the 45% possibility of occasions observed in the transfusion [22]. HU treatment was originally promising for additional stroke prevention, and the Stroke with Transfusions Changing to Hydroxyurea (SWiTCH) trial randomized participants with a stroke background to either pursuing chronic transfusion with iron chelation or HU with phlebotomy. It was concluded that transfusion/chelation remains the best way to manage children with SCA and stroke [29]. The first stroke was reported to occur when seven years old. Stroke was more common in serious genotypes, and ischemic stroke was more common compared to hemorrhagic stroke at this age [40]. HU treatment results in beneﬁcial hematological and biochemical impacts that resemble those observed in participants in chronic transfusion programs and which are most likely to add to improved blood ﬂow in vulnerable analytical vessels [40]. These impacts include raised hematocrit, lowered sickling, red cell adhesion, improved red cell rheology, and lowered hemolysis. Although the system of activity of chronic blood transfusions in minimizing the risk of stroke in children with SCD is not well comprehended, the resemblance of the observed impacts recommends HU as a feasible replacement for chronic blood transfusions. However, there are a couple of records where HU was used as the single treatment to avoid stroke reoccurrence. Most records on the use of HU for additional avoidance have remained in patients who formerly began on transfusion programs but were reluctant to proceed with chronic transfusion, with fairly excellent outcomes reported.

It was shown that 90% of the participants revealed a surge in Hb degree after HU therapy, and 75% of those that had the available outcome of HbF degree revealed a substantial increase in HbF after HU therapy consistent with a reputable effect of HU in children and adult with SCD [45]. It was well established that a high HbF concentration minimizes the intensity of SCD by preventing the development of HbS polymers. Also, the MCV was raised after HU therapy in 80% of the participants [45]. MCV values increased in lockstep with HbF concentrations, making MCV a good affordable substitute for HbF during therapy. When MCV increases, it improves sickle erythrocyte hydration, which increases sickle cell deformability which further adds to the effect of HU in minimizing the regularity of painful crisis. It was shown that a regular transfusion program designed to maintain HbS values below 30% is practical and safe in children with SCD at high risk for stroke [31]. 

Children registered in TCD With Transfusions Changing to Hydroxyurea (TWiTCH) trial had elevated iron build-up in both the liver and spleen; most had iron deposition in the kidney and some also had pancreatic deposition. If the intensity of parenchymal iron deposition was unexpected, the heterogeneity is perhaps not because the liver, pancreatic, kidney, and spleen have various systems and amounts of iron uptake. Considerable iron amount within the organs is a concern of a large cohort of children with SCA who are receiving chronic transfusions for proven cerebrovascular conditions. TWiTCH trial outcomes document the effectiveness of HU treatment for a cohort of children with SCA at high risk for primary stroke [46]. Particularly, children on the alternative arm that received an overlap duration with transfusions until attaining a steady HU maximum tolerated dose (MTD) efficiently maintained their average TCD rate throughout the study therapy duration. HU has numerous beneficial effects that should benefit children with SCA and irregular TCD velocities. One of the most important benefits is HbF induction, which decreases the intracellular % of HbS, prevents sickle polymer development, and prolongs erythrocyte survival [46]. TWiTCH individuals had a durable therapy reaction to HU, reaching an average of 27% HbF at MTD. However, since swelling and endothelial vasculopathy are features of the cerebrovascular condition, the lowered leukocyte and reticulocyte matters from HU should also be useful. Managing transfusion-acquired iron overload is also difficult for children on chronic transfusion treatment. After getting to a constant HU MTD and discontinuing transfusions, individuals in the alternative arm received monthly healing phlebotomy to lower their iron worry. Chronic transfusions are suggested for children with SCA and irregular TCD velocities (≥200 cm/s) to provide help prevent the event of the main stroke [28]. Irregular TCD velocities (>200 cm/s) are associated with a high risk for stroke and require transfusion treatment to lower the risk of primary stroke. The objective is usually to maintain the HbS concentration <30%; however, this objective is often difﬁcult to attain in clinical practice. HU treatment can replace chronic transfusions to sustain TCD velocities and assist in the prevention of primary stroke in high-risk children with SCA, abnormal TCD velocities, and at least one year of transfusions who do not have MRA-defined severe vasculopathy [46]. In addition, continuous HU administration since childhood seems safe and effective for SCA [47]. Initial moderate fixed-dose HU therapy is equivalent to initial regular blood transfusion therapy and preferable to no therapy [48].

## Conclusions

Ninety percent of children with SCA and irregular TCD velocities can be efficiently protected against the first stroke using chronic transfusion treatment to maintain HbS concentrations lesser than 30%. Prevention of cerebrovascular conditions in children with SCA, preferably through very early treatment with disease-modifying treatment, is the supreme objective. Although hereditary versions may influence stroke sensitivity, most stroke occasions in children with SCA remain inexplicable. HU can currently be considered a substitute treatment for preserving TCD velocities and possibly preventing primary stroke in selected high-risk children with SCA.

## References

[1] Leikin SL, Gallagher D, Kinney TR, Sloane D, Klug P, Rida W (1989). Mortality in children and adolescents with sickle cell disease. Cooperative Study of Sickle Cell Disease. Pediatrics.

[2] Gray A, Anionwu EN, Davies SC, Brozovic M (1991). Patterns of mortality in sickle cell disease in the United Kingdom. J Clin Pathol.

[3] Serjeant GR (1993). The clinical features of sickle cell disease. Baillieres Clin Haematol.

[4] Ware RE, Zimmerman SA, Schultz WH (1999). Hydroxyurea as an alternative to blood transfusions for the prevention of recurrent stroke in children with sickle cell disease. Blood.

[5] Adams RJ, McKie VC, Carl EM (1997). Long-term stroke risk in children with sickle cell disease screened with transcranial Doppler. Ann Neurol.

[6] Balkaran B, Char G, Morris JS, Thomas PW, Serjeant BE, Serjeant GR (1992). Stroke in a cohort of patients with homozygous sickle cell disease. J Pediatr.

[7] Ohene-Frempong K, Weiner SJ, Sleeper LA (1998). Cerebrovascular accidents in sickle cell disease: rates and risk factors. Blood.

[8] Adams RJ, McKie VC, Hsu L (1998). Prevention of a first stroke by transfusions in children with sickle cell anemia and abnormal results on transcranial Doppler ultrasonography. N Engl J Med.

[9] Steinberg MH, Barton F, Castro O (2003). Effect of hydroxyurea on mortality and morbidity in adult sickle cell anemia: risks and benefits up to 9 years of treatment. JAMA.

[10] Charache S, Terrin ML, Moore RD (1995). Effect of hydroxyurea on the frequency of painful crises in sickle cell anemia. Investigators of the Multicenter Study of Hydroxyurea in Sickle Cell Anemia. N Engl J Med.

[11] DeBaun MR (2020). Initiating adjunct low-dose hydroxyurea therapy for stroke prevention in children with SCA during the COVID-19 pandemic. Blood.

[12] Ohene-Frempong K (1991). Stroke in sickle cell disease: demographic, clinical, and therapeutic considerations. Semin Hematol.

[13] Craft S, Schatz J, Glauser TA, Lee B, DeBaun MR (1993). Neuropsychologic effects of stroke in children with sickle cell anemia. J Pediatr.

[14] Cohen MJ, Branch WB, McKie VC, Adams RJ (1994). Neuropsychological impairment in children with sickle cell anemia and cerebrovascular accidents. Clin Pediatr (Phila).

[15] Russell MO, Goldberg HI, Hodson A, Kim HC, Halus J, Reivich M, Schwartz E (1984). Effect of transfusion therapy on arteriographic abnormalities and on recurrence of stroke in sickle cell disease. Blood.

[16] Howard J (2016). Sickle cell disease: when and how to transfuse. Hematology Am Soc Hematol Educ Program.

[17] Swerdlow PS (2006). Red cell exchange in sickle cell disease. Hematology Am Soc Hematol Educ Program.

[18] Wilimas J, Goff JR, Anderson HR Jr, Langston JW, Thompson E (1980). Efficacy of transfusion therapy for one to two years in patients with sickle cell disease and cerebrovascular accidents. J Pediatr.

[19] Wang WC, Kovnar EH, Tonkin IL (1991). High risk of recurrent stroke after discontinuance of five to twelve years of transfusion therapy in patients with sickle cell disease. J Pediatr.

[20] Ware RE, Steinberg MH, Kinney TR (1995). Hydroxyurea: an alternative to transfusion therapy for stroke in sickle cell anemia. Am J Hematol.

[21] Rosse WF, Telen MJ, Ware RE (1998). Transfusion Support for Patients With Sickle Cell Diseases. https://www.amazon.com/Transfusion-Support-Patients-Sickle-Disease/dp/1563951037#detailBullets_feature_div.

[22] Bernaudin F, Verlhac S, Arnaud C (2016). Long-term treatment follow-up of children with sickle cell disease monitored with abnormal transcranial Doppler velocities. Blood.

[23] Platt OS, Brambilla DJ, Rosse WF, Milner PF, Castro O, Steinberg MH, Klug PP (1994). Mortality in sickle cell disease. Life expectancy and risk factors for early death. N Engl J Med.

[24] Higgins JPT, Thomas J, Chandler J, Cumpston M, Li T, Page MJ, Welch VA (editors) (2022). Cochrane Handbook for Systematic Reviews of Interventions. http://www.training.cochrane.org/handbook.

[25] Fridlyand D, Wilder C, Clay EL, Gilbert B, Pace BS (2017). Stroke in a child with hemoglobin SC disease: a case report describing use of hydroxyurea after transfusion therapy. Pediatr Rep.

[26] Darshana T, Rees D, Premawardhena A (2021). Hydroxyurea and blood transfusion therapy for sickle cell disease in South Asia: inconsistent treatment of a neglected disease. Orphanet J Rare Dis.

[27] Abboud MR (2020). Standard management of sickle cell disease complications. Hematol Oncol Stem Cell Ther.

[28] Aygun B, Wruck LM, Schultz WH (2012). Chronic transfusion practices for prevention of primary stroke in children with sickle cell anemia and abnormal TCD velocities. Am J Hematol.

[29] Aygun B, McMurray MA, Schultz WH (2009). Chronic transfusion practice for children with sickle cell anaemia and stroke. Br J Haematol.

[30] Brousse V, Hertz-Pannier L, Consigny Y (2009). Does regular blood transfusion prevent progression of cerebrovascular lesions in children with sickle cell disease?. Ann Hematol.

[31] Mirre E, Brousse V, Berteloot L (2010). Feasibility and efficacy of chronic transfusion for stroke prevention in children with sickle cell disease. Eur J Haematol.

[32] Franco JM, Borges CC, Ansaloni MA, Mauro RD, Souza YC, Braga JA (2020). Chronic transfusion therapy effectiveness as primary stroke prophylaxis in sickle cell disease patients. Hematol Transfus Cell Ther.

[33] Wood JC, Cohen AR, Pressel SL (2016). Organ iron accumulation in chronically transfused children with sickle cell anaemia: baseline results from the TWiTCH trial. Br J Haematol.

[34] DeBaun MR, Galadanci NA, Abdullahi SU (2014). Acceptability and safety of hydroxyurea for primary prevention of stroke in children with sickle cell disease in Nigeria. Blood.

[35] Ware RE (2010). How I use hydroxyurea to treat young patients with sickle cell anemia. Blood.

[36] Mulaku M, Opiyo N, Karumbi J, Kitonyi G, Thoithi G, English M (2013). Evidence review of hydroxyurea for the prevention of sickle cell complications in low-income countries. Arch Dis Child.

[37] de Montalembert M, Brousse V, Elie C, Bernaudin F, Shi J, Landais P (2006). Long-term hydroxyurea treatment in children with sickle cell disease: tolerance and clinical outcomes. Haematologica.

[38] Núñez RM, Figueroa CA, García-Perdomo HA (2020). Hydroxyurea can be used in children with sickle cell disease and cerebral vasculopathy for the prevention of chronic complications? A meta-analysis. J Child Health Care.

[39] Sumoza A, de Bisotti R, Sumoza D, Fairbanks V (2002). Hydroxyurea (HU) for prevention of recurrent stroke in sickle cell anemia (SCA). Am J Hematol.

[40] Ali SB, Moosang M, King L, Knight-Madden J, Reid M (2011). Stroke recurrence in children with sickle cell disease treated with hydroxyurea following first clinical stroke. Am J Hematol.

[41] Mvalo T, Topazian HM, Kamthunzi P (2019). Real-world experience using hydroxyurea in children with sickle cell disease in Lilongwe, Malawi. Pediatr Blood Cancer.

[42] Cunningham-Myrie C, Abdulkadri A, Waugh A, Bortolusso Ali S, King LG, Knight-Madden J, Reid M (2015). Hydroxyurea use in prevention of stroke recurrence in children with sickle cell disease in a developing country: a cost effectiveness analysis. Pediatr Blood Cancer.

[43] Lefèvre N, Dufour D, Gulbis B, Lê PQ, Heijmans C, Ferster A (2008). Use of hydroxyurea in prevention of stroke in children with sickle cell disease. Blood.

[44] Abdullahi SU, Jibir BW, Bello-Manga H (2020). Randomized controlled trial of fixed low-vs moderate-dose hydroxyurea for primary stroke prevention in Sub-Saharan Africa: final results of the spring trial. Blood.

[45] Al Hawsawi ZM, Ahmed Turkistani W (2008). Effect of hydroxyurea in children with sickle cell disease in Saudi Arabia. J Taibah Univ Medical Sci.

[46] Ware RE, Davis BR, Schultz WH (2016). Hydroxycarbamide versus chronic transfusion for maintenance of transcranial doppler flow velocities in children with sickle cell anaemia-TCD With Transfusions Changing to Hydroxyurea (TWiTCH): a multicentre, open-label, phase 3, non-inferiority trial. Lancet.

[47] Hankins JS, Aygun B, Nottage K, Thornburg C, Smeltzer MP, Ware RE, Wang WC (2014). From infancy to adolescence: fifteen years of continuous treatment with hydroxyurea in sickle cell anemia. Medicine (Baltimore).

[48] Galadanci NA, Abdullahi SU, Ali Abubakar S (2020). Moderate fixed-dose hydroxyurea for primary prevention of strokes in Nigerian children with sickle cell disease: final results of the SPIN trial. Am J Hematol.

